# Effectiveness of educational intervention and cognitive rehearsal on perceived incivility among emergency nurses: a randomized controlled trial

**DOI:** 10.1186/s12912-022-00930-1

**Published:** 2022-06-14

**Authors:** Shohreh Kousha, Ali Shahrami, Mohammad Mehdi Forouzanfar, Neda Sanaie, Foroozan Atashzadeh-Shoorideh, Victoria Skerrett

**Affiliations:** 1 Student Research Committee, School of Nursing and Midwifery, Shahid Beheshti University of Medical Sciences, Tehran, Iran; 2 Department of Emergency Medicine, School of Medicine, Imam Hossein Hospital, Shahid Beheshti University of Medical Sciences, Tehran, Iran; 3 Department of Emergency Medicine, School of Medicine, Shohadaye Tajrish Hospital, Shahid Beheshti University of Medical Sciences, Tehran, Iran; 4 Department of Medical-Surgical Nursing, School of Nursing and Midwifery, Shahid Beheshti University of Medical Sciences, Tehran, Iran; 5 Department of Psychiatric Nursing and Management, School of Nursing and Midwifery, Shahid Labbafinezhad Hospital, Shahid Beheshti University of Medical Sciences, Vali Asr Ave., Niayesh Cross Road, Opposite to Rajaee Heart Hospital, Tehran, 1996835119 Iran; 6 Mental Health Nursing, Birmingham City University, Birmingham, UK

**Keywords:** Incivility, Nursing, Workplace violence

## Abstract

**Background:**

Witnessing or experiencing of incivility affected the nurses’ perception of the ethical climate and quality of their work life. The aim of this study was to investigate the effectiveness of educational intervention and cognitive rehearsal on perceived incivility among emergency nurses.

**Method:**

This study was conducted as a randomized controlled parallel group clinical trial. Eighty emergency nurses participated in this study and were randomly assigned to intervention and control groups during December 2019—March 2020. Cognitive rehearsal program (include of definitions of incivility, ten common incivilities and appropriate practice methods for responding to each and role-plays) was delivered in five two-hour sessions over three weeks on different working days and shifts. The control group received only written information about what incivility is and how to deal with it before the implementation of intervention and one month after the completion of the training sessions, the demographic information form and the incivility scale were completed by the nurses.

**Results:**

The results showed that there was a significant effect on overall incivility, general incivility, and supervisor incivility between the intervention and control groups. However, these significant reductions were seen in control group who received only written education. There were no significant differences in nurse's incivility towards other nurses, physician incivility, and patient/visitor incivility between the two groups.

**Conclusion:**

The cognitive rehearsal program did not decrease perceived incivility among emergency department nurses in the short term.

**Trial registration:**

Our research was registered on clinicaltrials.gov. Registration number: IRCT20200714048104N1, first registration 16/07/2020.

## Introduction

Incivility is a dysfunctional interaction that is a recognized problem in the nursing workplace [1]. Nationally, 50.10% of Iranian nurse’s experience incivility [2]. Incivility is verbal and nonverbal behaviors that demean, reject, or exclude the person [3]. Witnessing or experiencing incivility affects nurses’ perceptions of the ethical climate and the quality of their work life [4].

There is a high level of incivility in the emergency department (ED) [5]. 62.6% of ED nurses reported that they had experienced uncivil behavior in the workplace. The most commonly perceived rude behaviors among ED nurses are blaming others for their mistakes or misdemeanors, displaying offensive body language, badmouthing others in the workplace, and gossiping about each other [6]. The consequences of these behaviors are low self-esteem, anxiety, sleep disturbances, recurrent nightmares, and depression in nurses [5]. Another consequence is also that of turnover intention [7, 8]. The prevalence of incivility in the emergency department is significantly related to nurse stress and burnout [9].

Nowadays, it is obvious that any kind of disrespect and incivility can change the organizational climate and increase the severity of burnout among nurses, lead to negative consequences such as clinical errors, and thus threaten patients' lives. Therefore, it is necessary to study how to deal with these behaviors [10].

One hypothesis for the continued prevalence of incivility in nursing is that nurses are socialized to maintain uncivil behaviors when a culture of incivility exists in nursing practice or academia [11]. This assumption is congruent with the underlying premise of Bandura's social learning theory. Bandura's social learning theory is a cognitive-behavioral approach that leads to behavior change [12]. The underlying premise of this model of nursing incivility is that nurses use social information from their environment as determinants of their behavior [13].

Some studies have hypothesized that the use of a series of interventions based on Bandura's social learning theory may help nurses cope with experienced incivility [14, 15]. Rehearsals based on this theory have been suggested in many studies to prepare nurses to recognize, deal with, and reduce incivility in education and the workplace [11, 16, 17], and to provide opportunities for appropriate behavior-based communication for future interactions [18].

In some studies, all participating nurses reported feeling able to recognize, cope with, and manage these behaviors after the cognitive rehearsal program [11, 19]. Some other studies believed that the cognitive rehearsal has impacts on the nurses' behavior over a long time. For example, in one study, 70% of participating nurses reported that they changed their behavior in bullying situations and that the occurrence of incivility decreased in the six months following training [20].

Although it appears that training and use of cognitive programs improves nurses' awareness of incivility and increases their ability to manage these behaviors and reduce the incidence of incivility, these changes vary across studies. The findings of a non-statistically significant decrease in the occurrence of incivility and a slight increase in nurses' awareness of incivility following the cognitive training programs are examples of these findings [21]. A culture-based study assumed that raising nurses' awareness of violence would lead to better recognition of these situations and that they would have to deal with more complex situations [7].

Given the commonality of the lived experience of incivility among Iranian nurses [22] and the need to reduce and prevent the consequences of these growing phenomena, especially in emergency departments as frontline services, this study hypothesizes that training and cognitive rehearsal will improve nurses’ ability to manage incivility in emergency departments. The aim of this study was to determine the effectiveness of educational intervention and cognitive rehearsal on perceived incivility among emergency nurses.

## Data and methods

### Study design and setting

This study was a single-blinded, parallel randomized clinical trial with two intervention and control groups. The codes and outcomes of the groups were blinded to practically and effectively reduce interpretation bias by the investigators.

The setting of the study was the emergency departments of two public hospitals affiliated Shahid Beheshti University of Medical Sciences in Tehran, Iran. 

### Participants

The study population consisted of the nurses employed in the inpatient departments of the emergency rooms (*N* = 120) of two public hospitals A and B of Shahid Beheshti University of Medical Sciences in Tehran, Iran, between December 2019 and March 2020.

#### Eligibility criteria 

Of 120 nurses, 106 nurses met all inclusion criteria through a short online questionnaire. Of the total 120 nurses, 56 nurses were enrolled in the emergency department of hospital A and 50 nurses were enrolled in the emergency department of hospital B. The two hospitals were randomly divided into an intervention group (Hospital A) and a control group (Hospital B) by tossing a coin. 

#### Inclusion criteria

The inclusion criteria for the nurses were having a bachelor's degree in nursing or higher, being a nurse working in the emergency departments of hospital A or B, willingness to participate in the study, and that they had not received any education or cognitive rehearsal based on Bandura's theory in the previous six months. 

#### Exclusion criteria

**Fig. 1:**
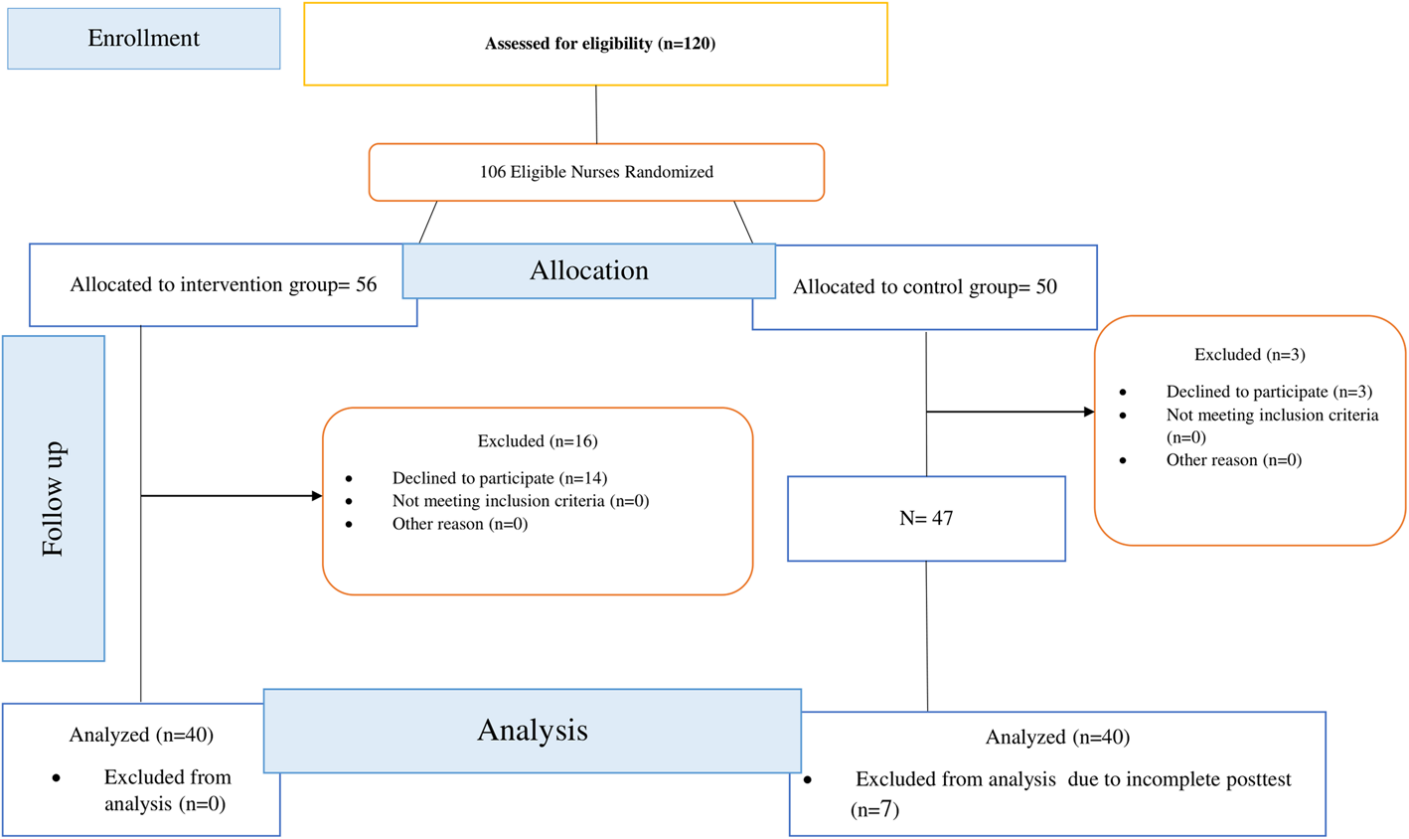
Flow diagram of the progress through the phases of two-group parallel randomized trial

The exclusion criteria were nurses who did not attend the first education session, who did not attend two education sessions, who did not complete the research instrument, and who did not complete the cognitive rehearsal. In Hospital A, 16 individuals dropped out during the study or did not participate in more than two sessions, leaving only 40. Of the 50 nurses from Hospital B, only 47 completed the scales, and 7 scales were completed incorrectly or incompletely, leaving 40 scales for analysis (Fig. 1).

### Sample size

Sample size was calculated based on results of Alshehry's study (2019) measuring the incivility using the Guidroz Nursing Incivility Scale (S^2^ = 0.64) [23], and with assuming an acceptable error of less than 0.05, a 95% confidence interval, and 80% test power through a formula with at least 41 samples in each group. Allowing for a 10% sample attrition, the final number of 92 samples was calculated. Revisiting the sample size with the total scores of incivility at posttest with a two-tailed independent t-test (t = 0.34) and a sample size of 40 in each group shows an effect size of 0.73, which is a medium to large effect size.

### Intervention 

The intervention group received the cognitive rehearsal program and the control group received only the written instructional material. The difference between educational programs in two groups is related to the nature of cognitive rehearsal. Cognitive rehearsal has a three-part didactic process that involves learning and rehearsing specific phrases to use during uncivil encounters [24]. 

#### Intervention group 

Cognitive rehearsal program (include of definitions of incivility, ten common incivilities and appropriate practice methods for responding to each and role-plays) was delivered in five two-hour sessions over three weeks on different working days and shifts. The intervention was delivered by an emergency nurse and a mental health nurse to make the program scenarios more professional. The content validity of the program was verified using the opinion of a 5-member expert panel (Kappa’s agreement coefficient = 0.80) and the standards of Kile's study [11].

The first session of the training covered definitions of incivility, its impact on nurses, patient safety, and the organization as a whole. The second session taught ten common incivilities and appropriate practical methods for responding to each. Role plays were conducted in the third, fourth and fifth sessions of the training. The interventions and sessions took place in the training classes of the hospital (Table 1).

**Table 1:** Content of cognitive rehearsal and educational sessions in a study on its effect of nurses’ perception of incivility

**Sessions**	**Component of cognitive rehearsal and educational sessions**
1	- Definitions of incivility behavior - Its effects on nurses, patient safety and organization
2	- Discussion of the cognitive rehearsal technique
	- Training on ten common incivility behavior and appropriate cognitive methods to respond to each of them (Table 2)
	- Demonstration scenarios
	- Practice scenarios
3	- Role play& feedback
4	- Role play& feedback
5	- Role play& feedback

Scenarios were distributed to the participants consisting of 10 common types of specific incivility, which were used following Kile's study [11], along with practical information and appropriate rehearsal behavioral methods to respond to and manage incivility (Table 2). After this step, the researchers and one of the participants performed the first two scenarios in front of the group. The participants were then divided into two small groups and asked to go through all the scenarios. Participants were then asked to apply these methods in their daily work.

**Table 2:** Cognitive rehearsal program contents (10 common types of specific incivility)

**Session**	**Topics**	**Scenario**	**Methods**
1	Communication without incivility	Instruction for cognitive practice and communication without incivility	Lecture
2	Nonverbal allusion	The nurse asks another nurse for help calculating the medication, but her colleague, standing nearby grinning and rolling her eyes, rolls hers as well	Expression of scenario Communication Role playing Feedback and evaluation
3	Retention of information	The reporting nurse does not give the following shift nurse important patient care information	Expression of scenario Communication Role playing Feedback and evaluation
4	Weakening efforts and motivation	A senior nurse says to the new graduate nurse who keeps making mistakes, “I don’t think you’re cut out for nursing.”	Expression of scenario Communication Role playing Feedback and evaluation
5	Humiliate	A senior nurse notes every mistake a new nurse has made in the shift notes	Expression of scenario Communication Role playing Feedback and evaluation
6	Gossiping	A nurse is talking behind someone who is not present	Expression of scenario Communication Role playing Feedback and evaluation
7	Subversion	A nurse is busy with his car accident patient. His colleague refuses to answer the new nurse’s patient inquiry	Expression of scenario Communication Role playing Feedback and evaluation
8	Lack of respect for privacy	A nurse asks another colleague why he/she went to the manager’s office	Expression of scenario Communication Role playing Feedback and evaluation
9	Verbal disrespect	An experienced nurse says to a newly graduated nurse, “I really don’t know how you managed to graduate?”	Expression of scenario Communication Role playing Feedback and evaluation
10	Physical violence	The ward manager yells at her/his colleague for requesting the wrong medication for a patient and throws the medication at him/her	Expression of scenario Communication Role playing Feedback and evaluation

#### Control group 

In Hospital B, participants were asked to complete the Incivility Scale at the beginning of the study and after one month without intervention (they were only given written information about what incivility is and how to deal with it). For equity and ethical reasons, the control group was offered training and a cognitive exercise after the study was conducted.

### Data collection

Written informed consent was obtained from the selected nurses before completing the questionnaires at baseline. The nurses' right to autonomy was protected. That they could make an informed decision to voluntarily participate in the research because they were informed of the potential risks and benefits of the research. Anonymity was maintained by using codes in the questionnaires and not linking the subject's identity to personal responses. on the confidentiality of the nurses' dialogues during meetings about the problems of incivility they face in the workplace, they assured.

Thereafter, nurses in the Emergency Department of Hospital A participated in five two-hour sessions over three weeks on different work days and shifts. Prior to conducting the training program and one month after completing the training sessions, the nurses completed the Demographic Information Form and Incivility Scale. 

### Research tool

#### Incivility Scale

The Incivility Scale was originally developed by Guidroz (2010) [25]. This study used a modified version of the Scale with 37 items, which has been used in previous Iranian studies on nurses [26]. This scale includes five subscales: general incivility (8 items), nurse in civility (9 items), supervisor incivility (5 items), physician incivility (6 items), and patient/visitor incivility (9 items). The scale is scored on a five-point Likert scale ranging from 1 (strongly disagree) to 5 (strongly agree), with higher scores representing higher perceptions of incivility. The reliability and validity of this scale has been investigated in numerous studies in Iran. In the study conducted by Kalantari et al., Cronbach's alpha was reported to be 0.86 [27]. In studies conducted in Iran, the Cronbach's alpha coefficient of the scale and of each source of general incivility, nurse incivility, supervisor incivility, physician incivility, and patient/visitor incivility was 0.95, 0.86, 0.93, 0.92, 0.87 and 0.85, respectively [26]. The Cronbach's alpha coefficient of the scale was 0.84 at the current study. 

### Data analysis

Data analysis was performed in SPSS version 21. An independent t-test for homogeneity of groups after random assignment was performed for the demographic variables. Analysis of variance (ANOVA) was used to compare the responses of the two groups before and after the intervention. General incivility, supervisor incivility, and total incivility before intervention and after intervention in two groups were compared using analysis of variance (ANOVA) after adjusting for nurse age and work history. Comparison of overall incivility, supervisor incivility and total incivility for the two groups was analyzed using repeated measures ANOVA. The significance level was set at *p* < 0.05 for all analyzes performed.

## Results

There were 40 samples from each hospital whose statistical data were analyzed. The mean age of nurses in the intervention and control groups was 31.6 ± 5.5 and 29.1 ± 4.4 years, respectively. The majority of nurses in both groups were female. It was found that there was no significant statistical difference between the intervention and control groups in terms of mean age, emergency department work experience, and work hours (Table 3).

**Table 3:** Demographic and work variables of participants in intervention and control groups

**Variables**	**Intervention**	**Control**	***p*** **value**
	M ± SD	M ± SD	
Age (year)	31.6 ± 5.5	29.1 ± 4.4	t = 2.20 *P* = .31
Work experience (year)	79.0 ± 68.4	59.5 ± 46.2	t = 1.50 *P* = .14
Work experience in emergency wards (months)	63.2 ± 60.5	45.6 ± 41.5	t = 1.52 *P *= .13
Work hours in week	53.8 ± 13.6	55.8 ± 12.1	t = .62 *P* = .54

*M* Mean, *SD* Standard Deviation.^*^p < 0.05

ANOVA and repeated-measures ANOVA showed that overall incivility and supervisor incivility increased in the intervention group but decreased in the control group. However, there were differences in overall incivility between the two groups, but these changes showed only an insignificant decrease in the control group (Table 4).

**Table 4:** Total incivility and its sub-scales in in two groups after adjusted age and nurse work history

**Variables**	**Intervention**		**Control**		***p*** value*
	**Pre**	**Post**	**Pre**	**Post**	
	**Mean ± SD**	**Mean ± SD**	**Mean ± SD**	**Mean ± SD**	
General incivility	2.4 ± 0.8	2.4 ± 0.7	2.3 ± 0.8	2.2 ± 0.8	0.01
Nurses’ incivility towards other nurses	2.2 ± 1.0	2.3 ± 0.8	2.4 ± 0.9	2.2 ± 0.7	0.06
Supervisor incivility	2.0 ± 0.8	2.3 ± 0.7	2.3 ± 0.7	2.2 ± 0.8	0.01
Physician incivility	2.6 ± 0.8	2.4 ± 0.9	2.9 ± 0.9	2.6 ± 0.8	0.12
Patient/visitor incivility	2.9 ± 0.9	2.7 ± 0.9	2.7 ± 0.9	2.5 ± 0.9	0.17
Overall incivility	2.4 ± 0.7	2.4 ± 0.7	2.5 ± 0.7	2.4 ± 0.7	0.01

*p* value calculated by repeated measures using ANOVA after adjusted age and nurse work history

*M* Mean, *SD* Standard Deviation

^*^p < 0.05

**Fig. 2:**
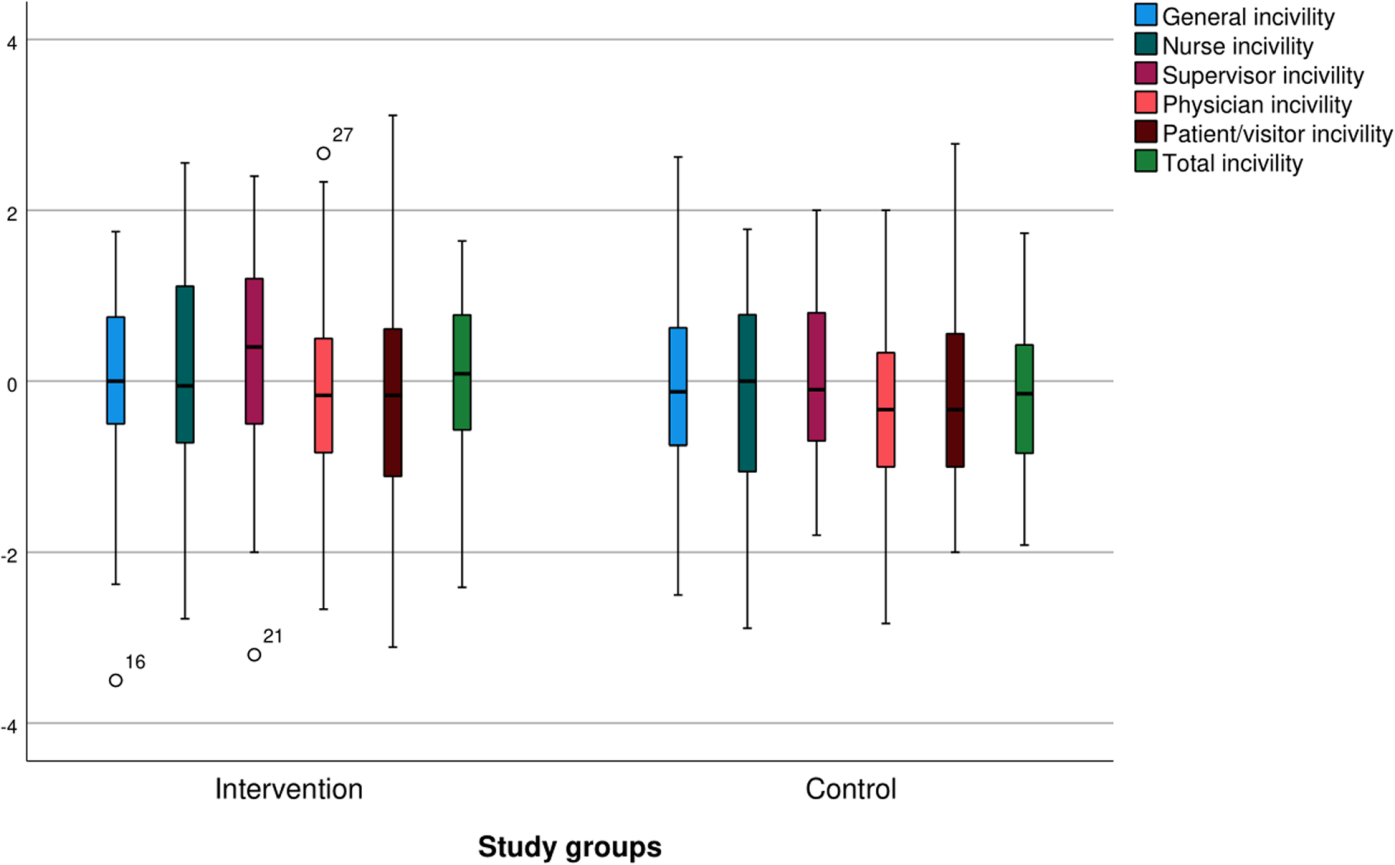
Changes in the incivility scores before and after the intervention between the two groups

Figure 2 shows the changes in the scores in the subscales of incivility and total incivility in the two groups of intervention and control. Although the figure shows that the scores of incivility in the control group generally decreased, the changes in both groups are almost the same, ranging from -1 to + 1 (2 scores).

## Discussion

The purpose of the current study was to determine the effectiveness of training and cognitive rehearsal on improving nurses’ ability to deal with incivility in emergency departments. One of the most effective methods to assist nurses in effectively responding to workplace incivility is the use of cognitive rehearsal [11]. Cognitive rehearsal involves mentally rehearsing responses to scenarios involving behaviors commonly associated with workplace incivility, such as nonverbal allusion, humiliation, gossiping, subversion, verbal disrespect, and physical violence. Cognitive rehearsal allows nurses to translate the training they received on effective communication techniques into acquired behaviors they can used in the workplace.

Results showed that the education and cognitive rehearsal had effects on general incivility, supervisor incivility, and overall incivility. After the intervention, overall incivility and supervisor incivility increased in the intervention group, while they decreased in the control group. Overall incivility showed significant differences between the two groups, with a significant decrease only in the control group, which received only written education. Patient or visitor incivility, physician incivility, and nurse’s incivility toward other nurses did not change significantly with the intervention. It may also be due to a paradoxical effect of workplace violence interventions. Some studies assumed that after an intervention, by increasing participants' awareness of workplace violence characteristics, a higher prevalence of workplace violence was recorded [28]. Another reason for this change in the present study could be the change in emergency management in the control group that occurred during the study, and it seems that the new leadership model has played an effective role in reducing incivility.

In the study by Kang et al. (2017), the cognitive rehearsal program did not reduce the bullying behaviors among nurses. They attributed this to the fact that the program focused only on nurses and did not address organizational issues, as well as the very early measurement of outcome variables [29].

It is hypothesized that the non-significant subscales of incivility, particularly patient or visitor incivility, and physician incivility are dependent on other structural causes inherent in nursing workplaces. Hosseinpour-Dalenjan et al. (2019) showed that there is a high correlation between nurses' work engagement and patient or visitor incivility [26]. Charrier et al. (2021) showed that nurses in the emergency department face lack of civility and deviant behaviors with patients and their accompanying family members, which requires strategies to manage the interpersonal difficulties [30]. An Iranian study showed that the incivility of physicians in the nursing workplace was the highest and the incivility of supervisors was the lowest [27]. Some studies assumed that incivility among emergency room physicians is high and is especially perceived in critical situations [31].

Clark et al. (2013) study showed that nurses experienced incivility before and after the intervention and also indicated that they tried to control their body language, tried to reduce or stop arguments with a colleague, and were aware of their behavior and reactions [32]. Warner et al. (2016) hypothesized that cognitive rehearsal has a greater effect in atmospheres with a higher prevalence of incivility [21].

It appears that the structural factors such as organizational culture are important in preventing and eliminating incivility in the nursing workplace [33]. The phrase “nurses eat their young” refers to the intentional mistreatment of nurses, by nurses. It is reported that more than one-third of nurses leave their jobs due to incivility [34].

The authors recommended that given the importance of incivility to patient, organizational, and caregiver outcomes, incivility in medical centers needs to be assessed regularly as a measure of organizational performance; and that necessary management actions should be taken based on the outcomes achieved. In this regard, it may be helpful to hold team meetings, address group dynamics, teach management skills, determine organizational values, recognize the organizational culture and history of organizational incivility as well as existing leadership techniques, and recognize the risks of incivility [33, 35]. Training on teamwork, appropriate communication, and managing incivility could be considered as complementary interventions for managing incivility among all health care healthcare professionals [35]. Also, modern innovative education approaches such as flipped classroom are crucial for nursing education, which leads to improving evidence-based teaching strategies and preparing nursing students for their future workplaces and proved to have positive effects in knowledge, risk assessment, and prophylaxis among nursing students [36]. This method can be recommended as an innovative and student-centered method in the teaching of evidence-based nursing practices for managing incivility in the workplace.

Education and cognitive rehearsal were effective in increasing nurses’ recognition of incivility and ability to confront it. Educational intervention improves civility in nursing practice and promotes a positive work environment in which patients are cared for safely and efficiently [11]. The use of workplace incivility education, training about effective responses to uncivil workplace behaviors, and active learning activities to practice newly learned communication skills will help nurses improve their ability to manage workplace incivility [37]. Workplace incivility has a negative impact on the health and well-being of health care team specially nurses. Long-term implementation of the cognitive rehearsal, training about effective responses to uncivil workplace behaviors, and the new leadership model can improve manage of incivility. Therefore, the need for the present study to help nurses to improve their ability to manage these types of behaviors seems necessary.

Our hypothesis about the effect of the training and cognitive rehearsal on improving nurses’ ability to deal with incivility in emergency departments is not confirmed by the findings of this study. Workplace incivility seems to be a multifaceted phenomenon that is affected by various variables such as time, gender, leadership model, organizational culture, etc. The study also has an important secondary finding, which is the mediated role of managers on perceived incivility among employees. In future studies, it is necessary to consider the mediated variables in the effectiveness of interventions affecting incivility.

## Limitations

The current study has some limitations, including the lack of blinding, which may cause the Hawthorne effect. The small sample size and focus on only nurses rather than all emergency professionals limit the generalizability of the results. Also, the changes in hospitals due to the pandemic COVID-19 could affect the results and led to a limitation in the long-term follow-up of participants. Another limitation of this study was the change in emergency department leadership in the control group during the study. Most of the participants in this study were women. The incidence and determinant of workplace incivility can differ by gender [38]. Future studies should ensure the recruitment of male participants to future investigate gender differences in the incidence of workplace incivility. This will provide data for the development of gender specific strategies to address incivility in the workplace.

The study contains some promising insights for the existing body of knowledge. It appears that the transformation of awareness of a relational problem into a behavioral reduction of that problem is more complex than it appears and may have a reverse effect. It is recommended that future studies be conducted to track the effects of the intervention over a three and six months’ period. Considering that only nurses participated in this study, it is recommended that training and sampling with nurses, physicians, and supervisors be conducted in future studies. Therefore, Future studies with longer interventions and higher frequent measurements are required to draw greater empirical conclusions regarding the outcome variables.

## Conclusion

The results of the study suggest that incivility in nursing is a widespread phenomenon in which several latent confounding variables that should be considered. Continuing education and cognitive rehearsal, greater emphasis on teamwork among nurses, physicians, nurse managers, and emergency department managers could prove effective if incorporated into continuing education, which warrants further study. However, the cognitive rehearsal program did not decrease incivility among emergency department nurses in the short time of our study. One reason for this could be the focus on individual levels and nurses, the other reason seems to be the limited time to measurement outcomes.

## Data Availability

All data generated or analyzed during this study are included in this published article and supplementary file.

## Abbreviations

ED: Emergency department
RCT: Randomized controlled trial
ANOVA: Analysis of variance
